# In Silico Analysis of the Effect of *Hydrastis canadensis* on Controlling Breast Cancer

**DOI:** 10.3390/medicina59081412

**Published:** 2023-08-02

**Authors:** Hima Vyshnavi AM, Sathianarayanan Sankaran, Krishnan Namboori PK, Baskar Venkidasamy, Abdurahman Hajinur Hirad, Abdullah A. Alarfaj, Ramachandran Vinayagam

**Affiliations:** 1 Computational Chemistry Group (CCG), Amrita School of Engineering, Amrita Vishwa Vidyapeetham, Coimbatore 641112, India; himaysh93@gmail.com; 2 Department of Pharmaceutical Chemistry, NGSM Institute of Pharmaceutical Sciences, Nitte (Deemed to be University), Deralakatte, Mangaluru 575018, India; ssnvij@yahoo.co.in; 3 Department of Oral & Maxillofacial Surgery, Saveetha Dental College and Hospitals, Saveetha Institute of Medical and Technical Sciences (SIMATS), Saveetha University, Chennai 600077, India; baskarbt07@gmail.com; 4 Department of Botany and Microbiology, College of Science, King Saud University, Riyadh 11451, Saudi Arabia; ahirad@ksu.edu.sa (A.H.H.); aalarfajj@ksu.edu.sa (A.A.A.); 5 Department of Biotechnology, Institute of Biotechnology, College of Life and Applied Sciences, Yeungnam University, 280 Daehak-Ro, Gyeongsan 38541, Gyeongbuk, Republic of Korea

**Keywords:** biomolecular networking, docking, pharmacokinetics, pharmacodynamics, triple-negative breast cancer, *Hydrastis canadensis*

## Abstract

*Background and Objectives:* Breast cancer is a significant type of cancer among women worldwide. Studies have reported the anti-carcinogenic activity of Hydrastis Canadensis (Goldenseal) in cancer cell lines. Hydrastis Canadensis could help eliminate toxic substances due to its anti-cancer, anti-inflammatory, and other properties. The design phase includes the identification of potential and effective molecules through modern computational techniques. Objective: This work aims to study Hydrastis Canadensis’s effect in controlling hormone-independent breast cancer through in-silico analysis. *Materials and Methods:* The preliminary screening of reported phytochemicals includes biomolecular networking. Identifying functionally relevant phytochemicals and the respective target mutations/genes leads to selecting 3D proteins of the desired mutations being considered the target. Interaction studies have been conducted using docking. The kinetic and thermodynamic stability of complexes was studied through molecular dynamic simulation and MM-PBSA/GBSA analysis. Pharmacodynamic and pharmacokinetic features have been predicted. The mechanism-wise screening, functional enrichment, and interactional studies suggest that canadaline and Riboflavin effectively interact with the target proteins. *Results:* Hydrastis Canadensis has been identified as the effective formulation containing all these constituents. The phytoconstituents; Riboflavin and Canadensis showed good interaction with the targets of hormone-independent breast cancer. The complexes were found to be kinetically and thermodynamically stable. *Conclusions:* Hydrastis Canadensis has been identified as effective in controlling ‘hormone-independent or basal-like breast cancer’ followed by ‘hormone-dependent breast cancer: Luminal A’ and Luminal B.

## 1. Introduction

Breast carcinoma, or breast cancer, has been reported as the second leading cause of death, next to lung cancer, especially among women [1]. The ‘multistep carcinogenesis’ and ‘tumour heterogeneity’ associated with transforming normal cells into cancer cells make monitoring and controlling the disease complex [2]. The cancer statistics in India during 2020 showed that the breast cancer incidence and mortality rate topped other cancer types, followed by cervical and ovarian cancers [3]. The ‘triple-negative breast cancer (TNBC)’, seen among 10–15% of patients, is the most challenging type of breast cancer and is characterised by a lack of expression of the estrogen receptor (ER), Progesterone receptor (PR) and human epidermal growth factor receptor-2 (HER2) [4]. Moreover, the TNBC mortality rate was about 40% during the initial five years from diagnosis [5]. TNBC has been identified as highly multi-targeted, necessitating monitoring, and controlling multiple pathways associated with the disease [6]. The ‘epidermal growth factor receptor (EGFR)’, an ErbB family of tyrosine kinase receptor proteins, has been found to be over-expressed in most of the (up to 78%) TNBC cases, suggesting it is a potential target for controlling the disease [7,8]. The TNBC pathway shows the involvement of three sub-pathways and biological processes in the development of the disease.

Biological functions result from molecular interactions, which are based on their structures. Molecular recognition, a dynamic process, could be made possible by docking either a small molecule or a protein molecule. The understanding of how a ligand bind to its target is a matter of concern in computational drug design [9]. Molecular interaction studies could be made possible through software such as Autodock, Glide, Gold, etc. [10,11,12]. In molecular docking, the critical step is identifying the target and ligand molecule. Functional prioritization of the target can be achieved by gene enrichment using machine learning approaches [13]. Several online tools and platforms, such as Toppgene, WebGIVI, and GORILLA, are available [14,15,16]. As most biological systems enjoy high smartness, a complete prediction of functionality based on any algorithm would be impossible. Hence, the identified genes after functional enrichment will be further prioritized using biomolecular networking and the associated information on molecular interactions [17]. The web-based networking platforms such as STRING provide the interactional and functional association between proteins, whereas STITCH (search tool for interactions of chemicals) focuses on the association between chemicals and proteins [18]. Cytoscape is a standard software program for visual biological networking and data integration [19]. The ‘protein–protein interaction network’ (PPIN) helps identify the most suitable target, while the ‘protein–drug interaction network (PDIN) determines the control molecules. Usually, the protein or drug with a maximum degree, closeness centrality and betweenness centrality will be selected as most relevant functionally and interaction-wise [17].

The ligands and targets obtained from the PDIN after docking have to be further justified by checking the kinetic stability of the protein–ligand complexes obtained after docking through ‘molecular dynamic (MD) simulation [20] and thermodynamic stability by performing the free energy evaluation through ‘Molecular Mechanics Poisson–Boltzmann surface area (MMPBSA)’ and ‘Molecular Mechanics Generalized Born surface area (MMPBSA/MMGBSA) studies. These techniques estimate the free energy of the complexes on an ensemble representing the bound and unbound states [21]. Molecular dynamic (MD) simulations have evolved throughout the years. The technique has advanced from simulating several hundreds of atoms to macromolecular complexes [22,23]. MD simulation could be used to improve ligand efficiency, a critical step in lead optimization. This could be performed by identifying the significant interactions between a ligand and the target’s binding pocket to predict potential ligand poses [24]. The simulation codes that are mainly used are AMBER, CHARMM, GROMACS, and NAMD [25]. The free energy of ligand–target interactions can be estimated through MM/PBSA and MM/GBSA approaches based on molecular dynamic simulations of the receptor–ligand complex [21].

Though the molecules show good interaction and binding affinities, a significant problem associated with the prognosis of the disease is the resistance to drug action developed by the body, especially during the prolonged use of the drugs [26]. Similarly, the metastasis of the disease leading to the spreading of cancer from one organ to another is a significant concern to be addressed. The effect of natural dietary products on the development and progression of breast cancer has been indicated in experimental studies [27]. Phytochemicals such as Fisetin and quercetin have antimetastatic properties in TNBC cell lines [28]. Ginko, Goldenseal (*Hydrastis canadensis*), ginseng, garlic, Echinacea, aloe vera, and saw palmetto are some plants used in breast cancer treatment [29]. In vivo studies showed the selective cytotoxic effect of Hydrastis against hormone-dependent breast cancer by induction of apoptosis [28]. The influence of Hydrastis in controlling the over-expression of targets of hormone-independent breast cancer is not excavated. Thus, the manuscript focuses on studying the effect of *Hydrastis canadensis* in controlling hormone-independent breast cancer/TNBC through molecular docking and evaluation through MD simulation and MM/PBSA and MM/GBSA analysis. In addition, to identify the phytochemicals that interact with the TNBC targets.

## 2. Materials and Methods

### 2.1. Identification of Target Genes

Breast cancer pathway analysis was studied using the KEGG (Kyoto Encyclopedia of Genes and Genomes) pathway [30]. The genes involved in the breast cancer pathway were identified. The gene enrichment analysis was carried out using the TOPP gene suite [31]. The genes were then screened based on their association with each other. A protein–protein interaction network was generated to find the associated genes through a biomolecular networking platform, Cytoscape 3.9.0 [17]. The 3D protein structures of candidate genes thus prioritised were identified from the PDB (Protein Data Bank) [32]. These structures were considered targets for the analysis. The functional domains of target protein molecules were identified through PROSITE [33]. The residues within the domain region were considered as the targets’ binding sites.

### 2.2. Identification of Control Drugs

The anti-breast cancer drugs were identified by the National Comprehensive Cancer Network (NCCN) [34]. A PDIN was generated using Cytoscape 3.9.0 to identify the drugs interacting with the targets [17]. The PDIN was evaluated through an interaction study. The drugs that interact with the targets were identified and are considered control drugs. The control drugs’ physicochemical properties, mechanism analysis, and ADMEtox analysis were studied using Datawarrior [35]. The complexes were evaluated through Molecular dynamic simulation and MM-GBSA/PBSA analysis [21].

### 2.3. Optimization of the Docking Algorithm

Molecular docking of ligand and target molecules was carried out using AutodockVina, Gold, and Glide. The protein was prepared by removing ‘heteroatoms and water molecules’ and performing protonation and energy minimisation. The domain region of the protein was considered the active site. The receptor grid was generated around the active site. The ligand molecules were prepared by adding hydrogen atoms and a forcefield [10,11,12].

### 2.4. Identification of Potential Phytochemicals

Dr Duke’s database and PubChem were used to identify the phytoconstituents in the Hydrastis Canadensis [36,37]. A ligand library was created using these constituents. Primary screening of ligands was carried out using Biomolecular networking, where a PDIN was generated using Cytoscape 3.9.0 [17]. The PDIN gave the chemical associated with protein. The constituents that showed interaction with the protein molecule were screened and subjected to mechanism analysis using Way2Drug Pass Online [38,39].

### 2.5. Identification of Potential Phytochemicals

The molecules with anti-breast cancer and anti-TNBC mechanisms were filtered out. The PDIN could be evaluated through an interaction study. The phytochemicals that interact with the targets were identified. The control drugs’ physicochemical properties and ADMEtox analysis were studied [40,41].

### 2.6. Evaluation of Ligand–Target Complex

The ligand–target complexes could be evaluated by studying their kinetic stability through molecular dynamic simulation and thermodynamic stability through MM-GBSA/PBSA analysis. This was achieved by integrating the OpenMM engine, LEaP program, and Amber toolkit in Python. The program was run on the cloud using “Google Colab Pro” (with the following specifications: GPUs: K80, P100, T4, CPU: 2 × vCPU, and RAM: 32 GB) [42].

#### 2.6.1. Molecular Dynamic Simulation

The MD simulation was carried out using the Amber tool. A system was built using the ligand–target complex, where an orthorhombic simulation box was created. AMBER force field-GAFF2 was used to build ligand topology. The solvated model was then exposed to 0.15 M salt concentration. NVT (Number Volume and Temperature) and NPT (Number Pressure and Temperature) ensembles were used by setting the temperature as 310 K, pressure as 1 pa, number of runs as 50,000,000, and simulation time as 100 ns. The results were analysed using ‘Root Mean Square Deviation (RMSD)’ and ‘Root Mean Square Fluctuation (RMSF)’ plots [43].

#### 2.6.2. MM-GBSA and MM-PBSA Analysis

Here, the interaction energy and solvation-free energy for the complex, receptor, ligand, and the average results to estimate the binding free energy were calculated. While carrying out the binding energy using the MM-GBSA method and the MM-PBSA method, the following GB/SA input parameters, such as “OBC” models (igb = 2) and Salt concentration = 0.15, were used. The analysis was carried out using Amber tools. The analysis was carried out to find the ‘binding free energy’ of the complex at 10 ns time intervals for 100 ns after MD simulation [43].

## 3. Results

Among 61 unique compounds in *Hydrastis canadensis*, 36 with TNBC-specific activities are included in Supplementary Table S1.

### 3.1. Identification of Targets

**Figure 1:**
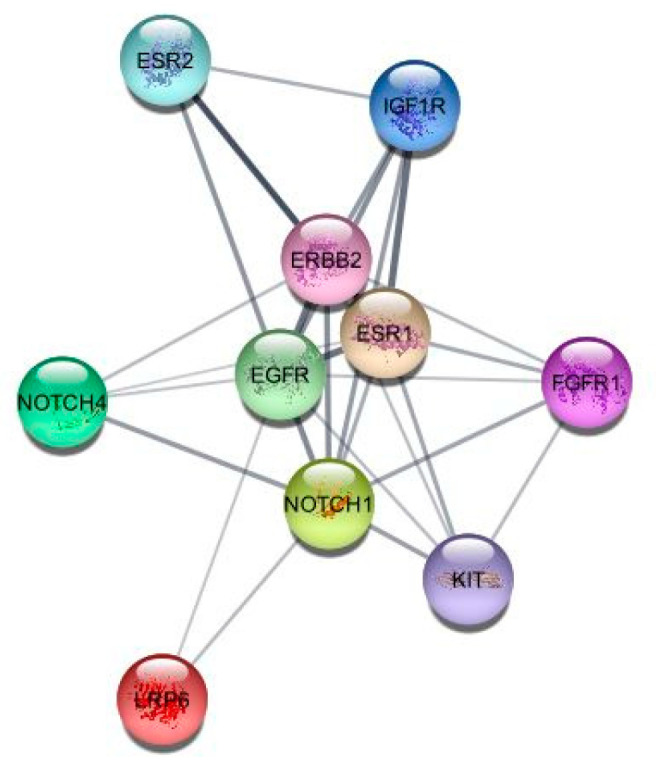
The protein–protein interaction network (PPIN).

The pathway analysis showed that the ErbB, Notch, and Wnt signalling pathways are involved in the ‘TNBC or basal-like breast cancer’. The PPIN analysis revealed ESR1, ESR2, PGR, EGFR, IGF1R, FGFR1, KIT, PTEN, mTOR, NOTCH 1, NOTCH 4, NFkB, CCND1, VEGFR3, LRP6, and APC (Table 1) as the candidate genes possessing the triple-negative breast cancer-specific functions and mechanisms. The PPIN obtained through bio-molecular networking is included in Figure 1.

All these genes kept a kinase domain. The 3D structures 1XKK (EGFR), 3LVP (IGF1R), 3MZW (ERBB2), and 1ERR (ESR1) were found in the kinase domain of their corresponding genes.

### 3.2. Identification of Control Drugs

The PDIN of approved anti-breast cancer drugs showed that Sorafenib, Dasatinib, Imatinib, Gefitinib, Sunitinib, Erlotinib, Vandetinib, Lapatinib, Pazopanib, Crizotinib, and Rapamycin had the highest degree above 20, highest closeness centrality, and highest betweenness centrality. Among them, Lapatinib exhibited an association with all the genes screened from the PPIN with an effective drug response of 3.897.

### 3.3. Optimization of the Docking Algorithm

Molecular docking with the approved kinase inhibitors Afatinib (D1), Dacomitinib (D2), Erlotinib (D3), Gefitinib (D4), Lapatinib (D5), Neratinib (D6), Poziotinib (D7), Sapitinib (D9), and Sunitinib (D10) using Glide and Autodock is shown in Table 2. Autodock gave good docking scores when compared to Glide and Gold scores. The complexes obtained from Autodock had good MMGBSA results. Lapatinib had a relatively high binding affinity among the kinase inhibitors with all the target molecules. Thus, Lapatinib was considered the control drug (Table 2).

### 3.4. Identification of Phytochemicals from Hydrastis canadensis

**Figure 2:**
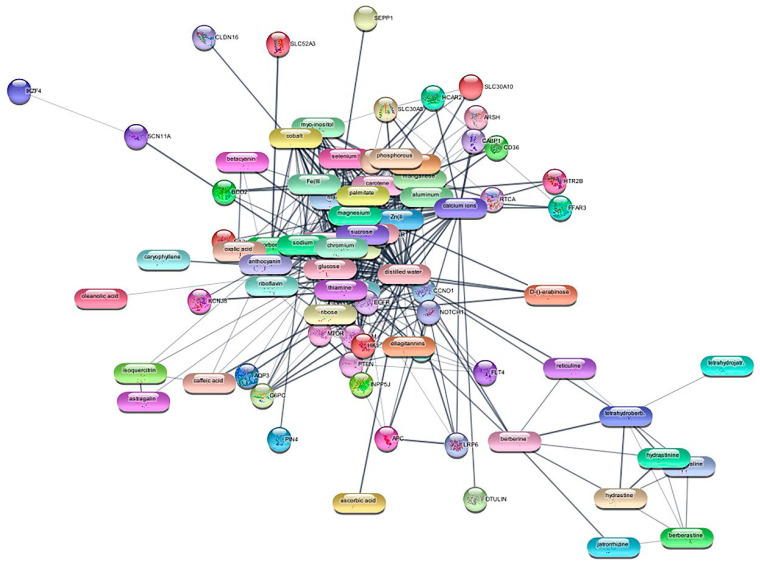
Protein–drug interaction network (PDIN) with phytochemicals.

A ligand library was created with the phytochemicals of Hydrastis Canadensis. The ligand library was subjected to an exhaustive screening technique where a PDIN was generated from the target genes and the ligands (Figure 2).

A total of 13 compounds had anti-breast cancer activity. The effective drug response of the molecules for TNBC-specific mechanisms is included in Figure S1. The physicochemical properties of the ligands were studied along with the ADMETox properties. The properties of molecules that followed Lipinski’s rule of five and had positive druglikeness areincluded in Supplementary Tables S2 and S3.

### 3.5. Molecular Docking

**Figure 3:**
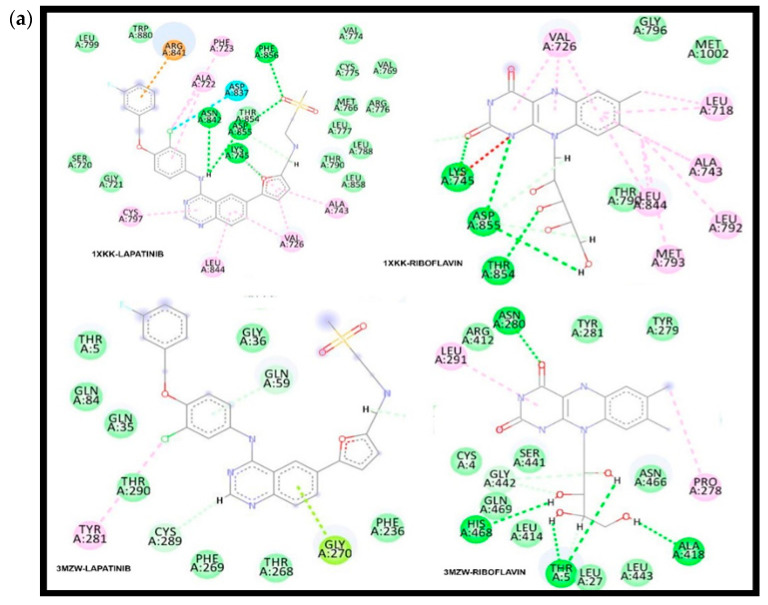

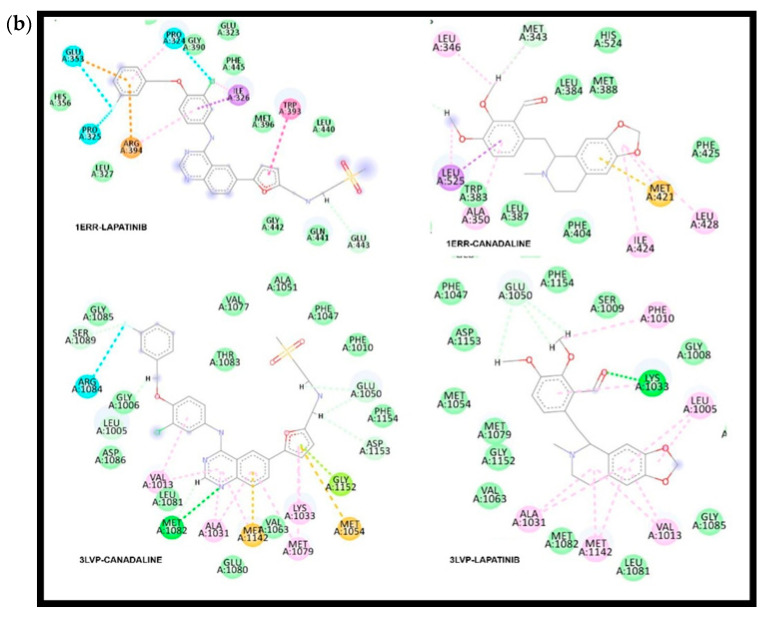
The ligand interaction diagram of the complexes. Where (**a**) includes 1XKK-Lapatinib, 1XKK-Riboflavin, 3MZW-Lapatinib, and 3MZ-Riboflavin, and (**b**) 1ERR-Lapatinb, 1ERR-Canadaline, 3LVP-Canadaline, and 3LVP-Lapatinb.

The molecules that passed the AMDETox analysis were subjected to the interaction study. The free binding energy of the control drug Lapatinib and phytochemicals is shown in Table 3. The ligand interaction diagram of the top interacting molecules is included in Figure 3.

#### 3.5.1. Molecular Dynamic Simulation

**Figure 4:**
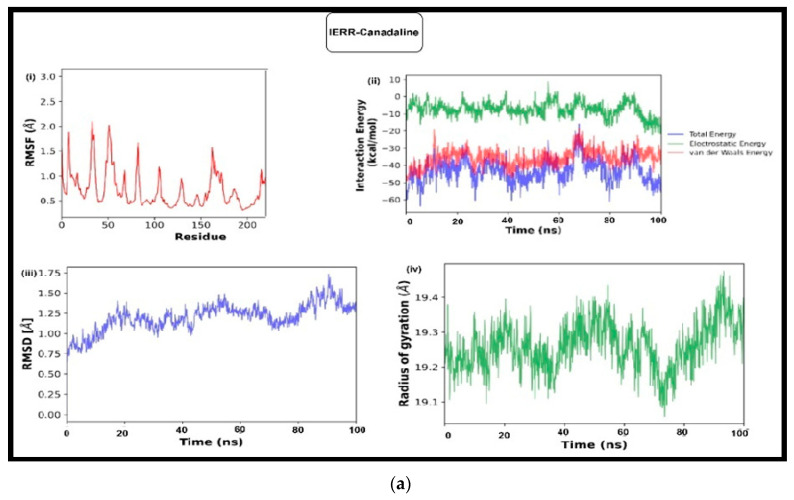

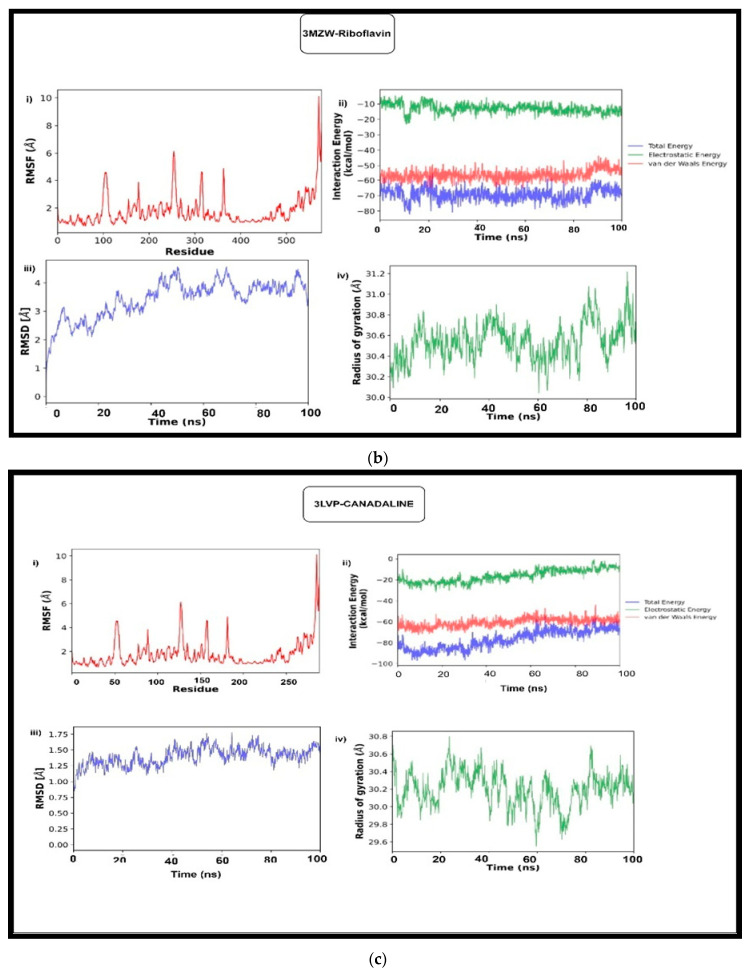

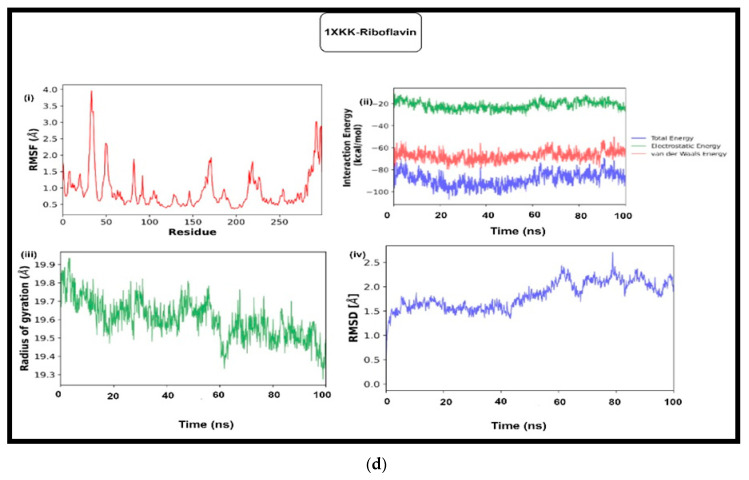
(**a**) MD plots (**i**) RMSF, (**ii**) hydrogen bond interaction, (**iii**) RMSD, and (**iv**) radius of gyration (Rg) of 1XKK-Riboflavin. (**b**) MD plots (**i**) RMSF, (**ii**) hydrogen bond interaction, (**iii**) RMSD, and (**iv**) radius of gyration (Rg) of 3LVP-Canadaline. (**c**) MD plots (**i**) RMSF, (**ii**) hydrogen bond interaction, (**iii**) RMSD, and (**iv**) radius of gyration (Rg) of 3MZW-Rioboflavin. (**d**) MD plots (**i**) RMSF, (**ii**) hydrogen bond interaction, (**iii**) RMSD and (**iv**) radius of gyration (Rg) of 1ERR-Canadaline.

The complexes 1XKK-Riboflavin, 3MZW-Riboflavin, 1ERR-Canadaline, and 3LVP-Canadaline were identified as kinetically stable across the analysis. The stability of the docked complexes was studied using RMSD and RMSF plots and the radius of gyration profiles (Figure 4a–d).

#### 3.5.2. MM-GBSA and MM-PBSA Analysis

The thermodynamic stability of the ligand–target complex was studied through MM-GBSA/PBSA analysis, which gave the Van der Waals, electrostatic energy, energy, polar energy, solvent accessible surface area, gaseous energy, solvation energy, and total energy for ligand, receptor, complex, and differences (complex–receptor–ligand). The docking_ΔGbind, MMGBSA_Δgbind, and MMPBSA_ΔGbind are included in Table 4.

**Figure 5:**
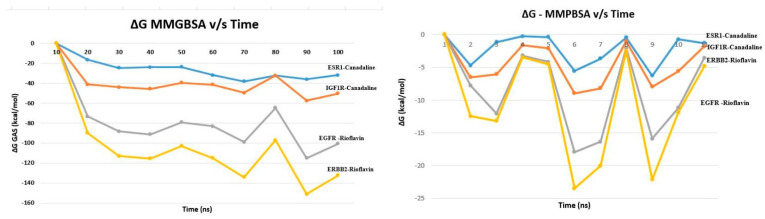
The ΔG_MMGBSA and ΔG_MMGPSA of the complexes.

While analysing the results for 10 ns intervals (Figure 5), it was found that although there were fluctuations in their values, they were all negative and thermodynamically stable. The study revealed variations in the MMGBSA_ΔGbind and MMPBSA_ΔGbind during different time intervals.

## 4. Discussion

The ErbB signalling pathway leads to proliferation, survival, and translocation; the Notch signalling pathway leads to NICD translocation, followed by homologous recombination, transcriptional regulation, and angiogenesis; and the Wnt signalling pathway leads to cell cycle progression [44]. The overexpression of the genes EGFR (Epidermal Growth Factor Receptor), KIT, and IGF1R leads to the activation of the ErbB signalling pathway where SHC-transforming protein 1 (SHC1), PI3KCA, GRB2, SC6, PTEN, AKT, RAS, RAF, MEK, mTOR, S6K, and ERK1/2 were found to be activated in the pathway [45]. The up-regulation of genes Notch1 and Notch 4 leads to the activation of the notch signalling pathway leading to NICD translocation, followed by the stimulation of genes such as HER2, Hey, NFkB, CCND1, and VEGFR3. The overexpression of LRP6 and FZD7 leads to the activation of the Wnt signalling pathway where PC, TCF, cMyc, and CCND1 are activated [46]. The estrogen signalling pathway leads to the over-expression of Estrogen receptors (ESR1 and ESR2), and the over-expression of Progesterone receptors (PGR) genes are involved in luminal A breast cancer. The MAPK and PI3K-A signalling pathways are found to be activated in Luminal B and HER2-positive breast cancer subtypes. In Luminal B, the hormone receptors are over-expressed, and the over-expression of HER2, FGFR1, and IGFR1 activates these pathways. In HER2-positive subtypes, the over-expression of genes HER2, EGFR, and IGF1R leads to the activation of signalling pathways [47]. The inhibition of mutations in candidate genes could lead to the inhibition of other mutations in the pathway.

Based on the shortest path, biological network analysis uses 11 topological methods, namely, Maximal Clique Centrality, Density of Maximum Neighbourhood Component, Maximum Neighbourhood Component, Edge Percolated Component, centralities (Bottleneck, Closeness, Stress, Radiality, Betweenness, and EcCentricity), and Degree [48]. The PPIN analysis is based on the centrality measures (betweenness, closeness, and degree). The degree of a node is one of the most frequently used metrics. The higher degree of a gene/protein suggests its key role in gene expression. The degree represents the neighbourhood of nodes. Studies showed that cancer genes/proteins show the highest degree of connectivity and are considered the hub node. The node closer to the hub node’s centre has a lower closeness centrality, indicating that it is more relative to its neighbouring nodes. Betweenness centrality gives the shortest routes passing through the nodes proportional to all other shortest paths connecting its adjacent nodes [49]. The network analysis showed that the gene EGFR had a more significant number of edges and was close to other network nodes. They were found to act as a bridge along the shortest path between the nodes. The genes EGFR, IGF1R, ERBB2, and ESR1 had degrees above 8.

The anti-breast cancer drugs such as Vemurafenib, Ponatinib, Crizotinib, Dacomitinib, Regorafenib, Pazopanib, Neratinib, Midostaurin, Nintedanib, Bosutinib, Sunitinib, Vandetanib, Dasatinib, Sorafenib, Lapatinib, Everolimus, Erlotinib, Temsirolimus, Gefitinib, Imatinib, and Rapamycin showed interactions with the genes EGFR and ERBB2. The evaluation of the PDIN showed that Sorafenib, Dasatinib, Imatinib, Gefitinib, Sunitinib, Erlotinib, Vandetinib, Lapatinib, Pazopanib, Crizotinib, and Rapamycin had the highest degree above 20, highest closeness centrality, and highest betweenness centrality. Among 27 drugs, Lapatinib was found to interact with all the target genes, and it was one of the drugs with the highest centrality measures and is considered as the hub node of the PDIN. The molecule showed anti-carcinogenic, antineoplastic, mTOR inhibition, and antimetastatic activity.

According to Dr Duke’s database, there were 61 phytochemicals in *Hydrastis canadensis*. The network analysis showed that 16 compounds interacted with the targets. The rest of the molecules were filtered out. The mechanism analysis of molecules gave the probability of activation, and the probability of inactivation of a molecule towards an activity [50]. All the phytoconstituents were found to possess antineoplastic (breast cancer, ovarian cancer, carcinogenic, and cervical cancer), antimetastatic, Transcription factor NF kappa B inhibitory, apoptosis agonist, and chemopreventive properties.

It was observed that molecules such as Berberastine, Berberine, Beta-Carotene, Chlorogenic Acid, and Jatrorrhizine had negative druglikeness; Beta-Carotene did not have ahydrogen bond acceptor or hydrogen bonding donor. The rest of the molecules followed Lipinski’s rule of five. It has been found that Alpha-Hydrastine, Ascorbic-Acid, Belladona Total Alkaloids, Canadaline, Canadine, Chlorogenic Acid, Corypalmine, Hydrastidine, and Riboflavin showed positive drug likeness (DL), Ligand Efficiency (LE), Lipophilic Ligand Efficiency (LLE), and LELP. They were non-mutagenic, non-tumorigenic, non-reproductive effective, and non-irritant.

They are soluble in water, BBB (Blood Brain Barrier) permeant, and showed high absorption. Riboflavin showed low absorption. Both Riboflavin and ascorbic acid were found to be non-BBB permeant in nature. These molecules were found to have inhibitory properties toward CYP enzymes (CYP1A2, CYP2C19, CYP2C9, CYP2D6, and CYP3A4). This suggests that the molecules are metabolised and excreted from the body through urine or sweat [51]. Canadine and Riboflavin form glycoprotein (Pgp) substrates, indicating their significant role excretion of metabolites through urine and bile, thereby preventing them from accumulating in the brain.

While optimizing the docking algorithms of Autodock and Glide, Autodock was found to be more suitable for the study. From the interaction study, it was observed that the phytochemicals showed good interaction with targets of ‘hormone-independent breast cancer or Luminal A’ than other breast cancer subtypes, ‘Luminal A or hormone-dependent breast cancer’. 1XKK-Lapatinib had three hydrogen bonding interactions between CL1, O5, and O6 atoms of ligand and OD1 of ASN842, N of ALA722, and NE of ARG841 within 3.72A, 2.90A, and 2.92A, making use of −1.00 kcal/mol, −4.10 kcal/mol, and −2.10 kcal/mol, respectively. 1XKK-C9 shared a pi-H bond between 6-ring and CG1 of amino acid VAL726 4.12A, using −0.7 kcal/mol energy. 3LVP-Lapatinib shared two hydrogen bonds with C13 and O6 atoms of the ligand molecule and ‘OE2 of GLU1050 and CE of LYS1033’ within 3.01A and 3.34A using −1.1 kcal/mol and −1.3 kcal/mol, respectively. 3LVP-C4 shared a hydrogen bond between the O5 atom of the ligand and N of SER1009 within 3.58A using −0.6 kcal/mol energy. While in complex 1ERR-C4, the O5 atom of the ligand molecule formed a hydrogen bond with the C atom of MET528 of the target within 3.69A using −0.90 kcal/mol. *Riboflavin* showed good interaction with the EGFR (1XKK) and ERBB2 (3MZW), and *Canadaline* showed good interaction with IGF1R (3LVP) and ESR1 (1EER).

The kinetic stability of ligand–target complexes was evaluated through molecular dynamic simulation. The mobility of protein segments was studied using the RMSF for each residue. The residues that showed fluctuations during MD simulation were identified from the RMSF value of the complex [52]. The residues 10LYS (1.50 Å), 40THR (4.00 Å), 60LEU (2.10 Å), 90ILE (2.40 Å), 160LYS (2.00 Å), and 290LYS (3.00 Å) of 1XKK-Riboflavin (Figure 4a(i)); 50ALA (5.00 Å), 100LYS (4.00 Å), 125MET (7.00 Å), 175GLY (~5.75 Å), and 290LYS (10.00 Å) of 3LVP-Canada line (Figure 4b(i)); 100PRO (5.00 Å), 250ALA (6.00 Å), 350PHE (5.75 Å), and 550LYS (10.00 Å) of 3MZW-Riboflavin (Figure 4C(i)); and 10VAL (1.90 Å), 30PRO (2.00 Å), 50HIS (2.00 Å), 160ARG (1.70 Å), and 220SER (1.30 Å) of 1ERR-Canada line (Figure 4d(i)) showed more fluctuations when compared to the other amino acid residues of the complexes.

The RMSD profile of 1XKK-Riboflavin (Figure 4a(iv)) was stable till 50 ns and tended to fluctuate. It fluctuated more between 60 ns and 85 ns and then tended to converge after 90 ns with an average value of ~2.0 Å. 3LVP-Canadaline (Figure 4b(iii)) showed a fluctuation till 80 ns with an average value of~1.50 Å, and showed a high fluctuation at 70 ns up to 1.75 Å. The RMSD profile of 3MZW-Riboflavin (Figure 4C(iii)) showed fluctuation till 70 ns. It converged after 80 ns with an average value of ~3.00 Å. Slight fluctuations were observed in the 1ERR-Canada line between 80 ns and 90 ns and then tended to converge at ~1.25 Å (Figure 4d(iii)) [53].

The ligand–target complexes were evaluated for the hydrogen bond stability of ligand molecules within the active site of the target molecule [54]. During MD (Molecular Dynamic) simulation, there was variation in the interaction between the ligand and the target complexes. 3LVP-C4 shared two new hydrogen bonds between C28 and O5 atoms of the ligand molecule and OE1 and CA atoms of GLU050 and GLN1007 within 2.98 Å and 2.53 Å, utilising −1.1 kcal/mol and −0.60 kcal/mol, respectively (Figure 4b(ii)). 3LVP-Lapatinib formed a pi-H bond between the 6-ring of the ligand molecule and the NE2 atom of GLN1007 within 4.68 Å, utilising −0.60 kcal/mol. The 1XKK-C9 complex formed a hydrogen bond between the O5 atom of the ligand and the NZ of LYS745 within 2.96 Å using −1.90 kcal/mol. 1XKK-Lapatinib is a hydrogen bond between the CL1 atom of the ligand molecule and the OD1 atom of ASN842 within 2.26 Å using −2.24 kcal/mol. The complex also had two pi–pi interactions between the 6-ring of the ligand and target molecules within 3.35 Å and 1.72 Å distance each. 1XKK-C9 lost the pi-H bond, and 3 H-bonds were created between O1NZ of amino acid LYS745, O3 of OG1 of amino acid THR854, and O6 of N atom of amino acid MET793 within 2.90 Å, 2.83 Å, and 3.41 Å, making use of −7.0 kcal/mol, −1.7 kcal/mol, and −2.1 kcal/mol energy, respectively, (Figure 4a(ii)).

The radius of gyration (Rg) is the product of the molecule’s mass and the ‘root means square distance’ of its number of atoms from the centroid. The minimum Rg value denotes the compactness of the complex. It shows whether the compound causes any modification in the secondary structure of the protein [55]. The sudden changes in the radius of gyration show interference of the compound in the structure of the protein (Figure 4a(iii),b(iv),c(iv),d(iv)). The complexes showedan average Rg of ~19.00 Å. Apart from the interactions, the convergence in the RMSF, RMSD, and Rg plots showed that these complexes were kinetically stable.

The MM-PBSA/GBSA analysis of the ligand–target complex has been studied to analyse the complex’s thermodynamic stability. The binding free energy (MMGBSA_ΔGbind and MMPBSA_ΔGbind) wascalculated by considering the energy terms such as Ebony (bond, angle, and dihedral), Eel (electrostatic), and EvdW (Van der Waals) interactions, Gopal (polar contribution), and Gnp (non-polar contribution), and the last term is T (absolute temperature) multiplied by S (entropy). Coulomb’s law was used to calculate the electrostatic term. To calculate the entropy term, all the water molecules and residues >8 Å were removed from the ligand. Non-polar solvation energy is in linear relation to the SASA (Solvent Accessible Surface Area) [56].

It was found that *‘Riboflavin-1XKK*, *Riboflavin-3MZW*, *Canadaline-3LVP*, and *Canadaline-1ERR’* are both kinetically and thermodynamically stable complexes.

## 5. Conclusions

The effect of *Hydrastis canadensis* in controlling breast cancer has been studied in this work. The gene enrichment analysis, functional enrichment, and pathway analysis showed that EGFR and IGF1R are genes at the initial phase of the TNBC-specific pathway, and ERBB2 and 1ESR are involved in the hormone-dependent breast cancer pathway. These genes were considered as the targets. Lapatinib has been identified as the control drug molecule through PDI network analysis. Among 61 phytochemicals from *Hydrastis canadensis*, Canadaline and Riboflavin were found to be potential molecules with anti-breast cancer properties. They satisfied physicochemical properties as well as the ADMETox properties. The in silico study showed that the phytochemical of *Hydrastis canadensis* had a good interaction score with both hormone-independent and hormone-dependent breast cancer. The complexes showed kinetic and thermodynamic stability. Thus, these molecules could be considered potential molecules that could down-regulate the breast cancer mutant genes, suggesting Hydrastins Canadensis’s role in controlling breast cancer subject to in vitro/vivo analysis.

## Figures and Tables

**Table 1:** PPI network analysis.

Genes	Betweenness	Closeness	Degree
**EGFR**	13	9	9
**IGF1R**	5	8.5	8
**ERBB2**	8.5	8.5	8
**ESR1**	5	8.5	8

**Table 2:** Optimization of docking algorithm.

Target	Parameters	D1	D2	D3	D4	D5	D6	D7	D8	D9
Hormone-Independent Breast Cancer Targets										
1xkk (EGFR)	Glide	−6.99	−7.45	−7.45	−8.68	−7.99	−8.39	−6.35	−7.87	−7.57
	Autodock	−8.39	−9.65	−7.88	−8.59	−9.57	−8.98	−8.94	−9.26	−7.96
	RMSD	2.36	1.54	1.51	2.37	2.06	1.98	2.37	1.70	3.09
	MMGBSA_ΔG	−57.18	−60.28	−51.47	−61.66	−57.51	−67.27	−47.17	−50.15	−40.43
	MMGBSA_ΔG_(NS)_	−63.00	−66.92	−59.53	−70.56	−63.55	−73.10	−52.79	−59.60	−51.12
3LVP (IGF1R)	Glide	−6.64	−7.31	−6.41	−4.62	−7.14	−6.24	−6.50	−7.91	−7.40
	Autodock	−7.51	−6.96	−7.07	−7.16	−7.69	−8.26	−7.27	−7.50	−6.81
	RMSD	0.949	1.59	1.27	2.30	1.18	3.47	2.86	2.58	2.03
	MMGBSA_ΔG	−31.48	−44.22	−48.01	−34.77	−45.00	−42.16	−52.71	−61.92	−53.57
	MMGBSA_ΔG_(NS)_	−39.72	−50.81	−58.40	−39.90	−56.14	−55.52	−55.9	−64.70	−64.36
Hormone-Dependent Breast Cancer Targets										
3MZW (ERBB2)	Glide	−5.04	−5.46	−2.93	−3.56	−5.69	−4.89	−3.49	−3.16	−3.99
	Autodock	−5.96	−6.08	−6.79	−6.25	−6.39	−7.00	−5.95	−6.08	−5.96
	RMSD	2.02	1.83	2.39	1.38	1.65	2.69	2.75	2.39	4.62
	MMGBSA_ΔG	−41.41	−41.38	−36.34	−27.72	−49.83	−44.73	−29.31	−24.42	−35.30
	MMGBSA_ΔG_(NS)_	−48.45	−48.43	−38.31	−31.82	−52.95	−47.80	−34.15	−30.26	−39.34
1EER (ESR1)	Glide	−7.16	−7.25	−7.84	−6.48	−6.94	−5.15	−5.28	−6.05	−5.25
	Autodock	−8.38	−8.03	−8.39	−7.35	−8.57	−7.78	−7.40	−7.63	−6.46
	RMSD	1.17	1.98	1.09	1.68	2.76	1.12	2.40	2.04	3.30
	MMGBSA_ΔG	−52.38	−51.17	−29.77	−56.45	−66.48	−45.21	−50.13	−39.92	−36.70
	MMGBSA_ΔG_(NS)_	−54.39	−56.67	−34.57	−58.99	−68.72	−51.86	−52.21	−49.03	−42.32

**Table 3:** The free binding energy of phytochemicals.

Target	D1	C1	C2	C3	C4	C5	C6	C7	C8	C9
	Hormone-Independent Breast Cancer Targets									
1XKK	−9.570	−6.060	−4.660	−4.710	−3.896	−5.980	−5.330	−5.950	−5.450	−8.280
3lvp	−7.690	−6.830	−6.240	−6.680	−7.060	−6.560	−6.170	−6.150	−6.060	−6.770
	Hormone-Dependent Breast Cancer Targets									
1ERR	−5.890	−6.070	−6.600	−5.10	−6.820	−5.840	−6.140	−5.970	−5.290	−6.750
3MZW	−6.330	−6.520	−6.820	−4.510	−6.000	−6.350	−5.960	−6.120	−6.500	−7.380

Alpha-Hydrastine (C1), Ascorbic-Acid (C2), Belladona Total Alkaloids (C3), Canadaline (C4), Canadine (C5), Chlorogenic Acid (C6), Corypalmine (C7), Hydrastidine (C8), Riboflavin (C9)’, control drugLapatinib (D1), and the target proteins.

**Table 4:** docking_ΔGbind (ΔG__D_), MMGBSA_ΔGbind (ΔG__G_), and MMPBSA_ΔGbind (ΔG__P_) in kcal/mol.

Target	1XKK			3LVP			3MZW			IERR		
Parameter	ΔG__D_	ΔG__G_	ΔG__P_	ΔG_D	ΔG_G	ΔG_P	ΔG_D	ΔG_G	ΔG_P	ΔG_D	ΔG_G	ΔG_P
**D1**	−9.57	−23.74	−0.36	−7.69	−32.11	−1.27	−5.890	−24.70	−1.34	−6.330	−24.08	−0.26
**C4**	−3.90	−24.82	−25.32	−7.06	−38.24	−5.55	−6.820	−15.89	−1.72	−6.000	−35.24	−3.67
**C9**	−8.28	−25.86	−24.95	−6.77	−31.94	−3.67	−6.750	−32.90	−0.49	−7.380	−31.94	−5.55

## Data Availability

From open-source software.

## Supplementary Materials

The following supporting information can be downloaded at https://www.mdpi.com/article/10.3390/medicina59081412/s1, and the following supporting information in Figure S1: The Effective drug response of the molecules towards TNBC specific mechanisms; Table S1: The unique phyto-constituents present in Goldenseal which showed some activity. Table S2: Drug likeliness and physichochemical properties of phytochemicals. Table S3: ADME properties of the phytochemicals.
