# Supplementary material for: In Silico Analysis of the Effect of Hydrastis canadensis on Controlling Breast Cancer
Source: Medicina (Kaunas). 2023 Aug 2;59(8):1412. doi: 10.3390/medicina59081412 (PMC10456556; doi:10.3390/medicina59081412)
Supplement: Supplementary file 1 [file medicina-59-01412-s001.zip › medicina-2464067-supplementary.pdf]

In Silico Analysis of the Effect of *Hydrastis canadensis* on Controlling Breast Cancer

Hima Vyshnavi AM [1], Sathianarayanan Sankaran [2], Krishnan Namboori PK [1,*], Baskar Venkidasamy [3], Abdurahman Hajinur Hirad [4], Abdullah A. Alarfaj [4] and Ramachandran Vinayagam [5,*]

1 Computational Chemistry Group (CCG), Amrita School of Engineering, Amrita Vishwa Vidyapeetham, Coimbatore 641112, India; himaysh93@gmail.com
2 Department of Pharmaceutical Chemistry, NGSM Institute of Pharmaceutical Sciences, Nitte (Deemed to be University), Deralakatte, Mangaluru 575018, India; ssnvij@yahoo.co.in
3 Department of Oral & Maxillofacial Surgery, Saveetha Dental College and Hospitals, Saveetha Institute of Medical and Technical Sciences (SIMATS), Saveetha University, Chennai 600077, India; baskarbt07@gmail.com
4 Department of Botany and Microbiology, College of Science, King Saud University, Riyadh 11451, Saudi Arabia; ahirad@ksu.edu.sa (A.H.H.); aalarfajj@ksu.edu.sa (A.A.A.)
5 Department of Biotechnology, Institute of Biotechnology, College of Life and Applied Sciences, Yeungnam University, 280 Daehak-Ro, Gyeongsan 38541, Gyeongbuk, Republic of Korea
* Correspondence: n_krishnan@cb.amrita.edu (K.N.P.); rambio85@gmail.com (R.V.)

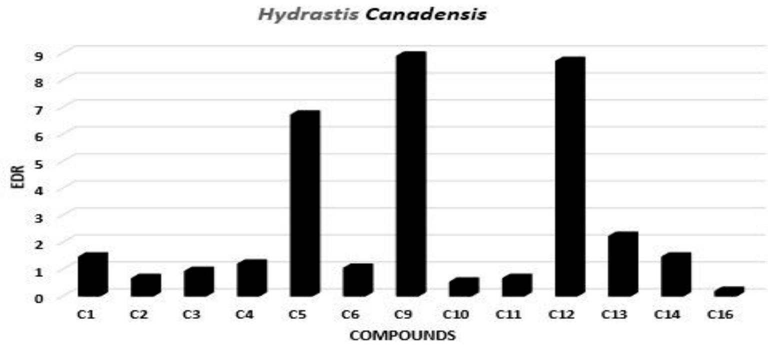

Figure S1. The Effective drug response of the molecules towards TNBC specific mechanisms

Table S1. The unique phyto-constituents present in Goldenseal which showed some activity

Chemical	Plant Part	Chemical	Plant Part
(-)-Alpha-Hydrastine	Plant	Inositol	Plant
(-)-Canadine	Plant	Iron	Root
Alpha-Hydrastine	Root	Jatrorrhizine	Root
Aluminum	Root	Magnesium	Root
Ascorbic-Acid	Root	Manganese	Root
Berberastine	Plant	Niacin	Root
Berberine	Shoot	Phosphorus	Root
Beta-Carotene	Root	Potassium	Root
Calcium	Root	Reticuline	Plant
Canadine	Root	Riboflavin	Root
Chlorogenic-Acid	Plant	Selenium	Root
Chromium	Root	Silicon	Root
Cobalt	Root	Sodium	Root

Corypalmine	Root	Starch	Root
D-Glucose	Plant	Sucrose	Plant
Fiber	Root	Thiamin	Root
Hydrastine	Plant	Tin	Root
Hydrastinine	Plant	Zinc	Root

Table S2. Drug likeliness and physichochemical properties of phytochemicals

Structure	MW	cLogP	cLogS	HBA	HBD	PSA	DL	LE	LLE	LELP
(-)-Alpha-Hydrastine	383.40	2.75	-3.38	7	0	66.46	4.34	0.16	0.59	16.76
(-)-Canadine	339.39	3.12	-3.67	5	0	40.16	2.81	0.26	1.56	12.13
(+)-Canadine	339.39	3.12	-3.67	5	0	40.16	2.81	0.18	0.24	16.91
Alpha-Hydrastine	383.40	2.75	-3.38	7	0	66.46	4.34	0.16	0.59	16.76
Ascorbic-Acid	176.12	-2.46	-0.35	6	4	107.22	0.02	0.14	3.73	-17.08
Total Alkaloids	542.72	5.18	-5.11	6	0	59.08	2.82	0.12	-1.60	42.23
Berberastine	352.37	-0.20	-4.12	6	1	61.03	-1.43	0.18	3.56	-1.15
Berberine	336.37	0.52	-4.67	5	0	40.8	-2.25	0.31	5.11	1.69
Beta-Carotene	536.89	13.87	-7.33	0	0	0 .00	-3.35	0.07	-11.60	177.62
Canadaline	369.42	3.56	-3.76	6	0	57.23	2.42	0.10	-1.55	34.92
Canadine	339.39	3.11	-3.67	5	0	40.16	2.81	0.24	1.35	12.71
Chlorogenic Acid	354.31	-0.70	-1.50	9	6	164.75	-0.98	0.15	3.51	-5.11
Corypalmine	341.41	2.58	-2.68	5	1	51.16	2.90	0.20	1.15	12.62
Hydrastidine	383.40	2.75	-3.38	7	0	66.46	4.34	0.18	0.95	15.18
Jatrorrhizine	338.38	-0.04	-3.68	5	1	51.80	-2.25	0.22	4.14	-0.021
Riboflavin	376.37	-2.07	-2.43	10	5	155.05	6.19	0.17	5.373	-12.30

Table S3. ADME properties of the phytochemicals

Structure	Aromatic heavy atoms	#Rotatable bonds	MR	Solubility	GI absorption	BBB permeant	Pgp substrate	CYP Inhibition
(-)-Alpha-Hydrastine	12	3	103.38	Moderately soluble	High	Yes	No	Yes
(-)-Canadine	12	2	97.07	Moderately soluble	High	Yes	Yes	Yes
(+)-Canadine	12	2	97.07	Moderately soluble	High	Yes	Yes	Yes
Alpha-Hydrastine	12	3	103.38	Moderately soluble	High	Yes	No	Yes
Ascorbic-Acid	0	2	35.12	Soluble	High	No	No	No
Total Alkaloids	12	7	162.51	Poorly soluble	High	Yes	No	No
Berberastine	16	2	96.04	Moderately soluble	High	Yes	Yes	Yes
Berberine	16	2	94.87	Moderately soluble	High	Yes	Yes	Yes
**Beta-Carotene**	0	10	184.43	Moderately soluble	High	Yes	Yes	Yes
Canadaline	12	5	104.41	Moderately soluble	High	Yes	Yes	No
Canadine	12	2	97.07	Moderately soluble	High	Yes	Yes	Yes
**Chlorogenic Acid**	6	5	83.5	Soluble	Low	No	No	No
Corypalmine	12	3	99.52	Moderately soluble	High	Yes	Yes	No
Hydrastidine	12	3	103.38	Moderately soluble	High	Yes	No	Yes
Jatrorrhizine	16	3	97.33	Moderately soluble	High	Yes	Yes	Yes
Riboflavin	14	5	96.99	Soluble	Low	No	No	No
